# Total mesorectal excision quality in rectal cancer surgery affects local recurrence rate but not distant recurrence and survival: population-based cohort study

**DOI:** 10.1093/bjsopen/zrae071

**Published:** 2024-08-08

**Authors:** Åsa Collin, Cecilia Dahlbäck, Joakim Folkesson, Pamela Buchwald

**Affiliations:** Department of Surgical Sciences, Uppsala University Hospital, Uppsala, Sweden; Department of Clinical Sciences Malmö, Lund University, Malmö, Sweden; Department of Surgery, Skåne University Hospital, Malmö, Sweden; Department of Surgical Sciences, Uppsala University Hospital, Uppsala, Sweden; Department of Clinical Sciences Malmö, Lund University, Malmö, Sweden; Department of Surgery, Skåne University Hospital, Malmö, Sweden

## Abstract

**Background:**

The quality of the total mesorectal excision specimen in rectal cancer surgery is assessed with a three-tier grade (mesorectal, intramesorectal and muscularis propria). This study aimed to analyse the prognostic impact of the total mesorectal excision grade on survival, and to identify risk factors for intramesorectal and muscularis propria resection in a population-based setting.

**Methods:**

All patients in the Swedish Colorectal Cancer Registry with rectal cancer stage I–III ≤ 10 cm from the anal verge, diagnosed 2015–2019, undergoing total mesorectal excision were analysed. Clinical, surgical and pathological data were retrieved and analysed for the following primary outcomes: local and distant recurrence and overall and relative survival; secondary outcomes were risk factors for total mesorectal excision grading (intramesorectal or muscularis propria resection). Of note, postoperative death < 30 days or recurrence within 90 days were exclusion criteria for survival and recurrence analysis. Recurrence-free patients with less than 3 years follow-up, and patients lacking data regarding recurrence, were also excluded from recurrence analyses.

**Results:**

Overall, of 7979 patients treated during the study interval, 1499 patients were eligible for recurrence, 2441 patients for survival and 2476 patients for risk-factor analyses, of which 75% were graded mesorectal, 17% intramesorectal and 8% muscularis propria. Median follow-up for survival was 42 (1–77) months. The worst total mesorectal excision grading (muscularis propria resection) was an independent risk factor for local recurrence in multivariable analysis (HR 2.73, 95% c.i. 1.07 to 7.0, *P* = 0.036). Total mesorectal excision grade had no impact on distant recurrence or survival. Female sex, tumour level <5 cm, abdominoperineal resection, minimally invasive surgery (laparoscopic and robotic), high blood loss, long duration of surgery and intraoperative perforation were independent risk factors for worse total mesorectal excision grading (intramesorectal and/or muscularis propria resection) in multivariable analyses.

**Conclusion:**

Muscularis propria resection increases the risk of local recurrence but does not seem to affect distant recurrence or survival.

## Teaser

This study aimed to analyse the prognostic value of the total mesorectal excision grade, and identify risk factors for intramesorectal and muscularis propria resection in a population-based setting. The primary outcomes were local and distant recurrence and overall and relative survival; secondary outcomes were risk factors for intramesorectal or muscularis propria resection. Muscularis propria resection increases the risk of local recurrence but does not seem to affect distant recurrence or survival.

## Introduction

Total mesorectal excision (TME) is the ‘standard’ surgical procedure for most rectal cancer patients, with a reported decrease in local recurrence rates from 25–40% to 5–10%^1–3^. The technique requires a precise dissection in the avascular plane between the presacral and mesorectal fascia, enabling excision of the entire rectum with surrounding lymphatic and venous drainage, preserving the mesorectal fascial envelope as an oncologic package^4^. In order to histologically assess the quality of the TME specimen, Quirke *et al.* defined a three-graded score, referred to as mesorectal, intramesorectal and muscularis propria, depending on what plane the dissection follows^5,6^. An association between the TME quality, according to this grading system, and local recurrence has been shown in several trials^6–12^.

Contradictory results have been presented regarding distant recurrence and survival in relation to TME quality^5,7,11,13^. Most studies have been retrospective with fairly small numbers of patients and many are outdated.

The primary aim of this study was to determine the prognostic value of TME quality on cancer recurrence and survival in a population-based setting. The secondary aim was to describe patient factors, tumour characteristics and treatment factors influencing the TME quality, to identify risk factors for intramesorectal and muscularis propria resections.

## Methods

### Study population

Data from patients diagnosed with rectal cancer between 1 January 2015 to 31 December 2019, who had undergone elective surgical intervention, were retrieved from the Swedish Colorectal Cancer Registry (SCRCR). Data included information on sex, age, body mass index (BMI), American Society of Anesthesiologists’ (ASA) score, tumour level, clinical and pathological stage, perioperative and histopathological data, and local and distant recurrence. The registration of rectal cancer in the SCRCR started in 1995 and has nearly 100% coverage^14^. The TME grade was included in the registry in 2015, which is why inclusion was set from this year.

To ensure that all included patients had undergone TME, only standard radical procedures for rectal cancer, anterior resection (AR), abdominoperineal resection (APR) or Hartmann’s procedure, with a tumour level ≤10 cm from the anal verge, were included. Patients with clinical stage IV cancer, non-radical (R1) resection or missing data regarding type of surgical procedure, tumour level, clinical stage or TME grade, were excluded. Patients were divided into three groups according to TME grade (mesorectal, intramesorectal and muscularis propria), and included in risk factor analyses for intramesorectal or muscularis propria resection. The study was approved by the Swedish Ethical Review Authority (2020-00981) and complies with the guidelines of the Declaration of Helsinki.

### Definitions

All included rectal cancers were adenocarcinomas. The level of the tumour was measured with rigid sigmoidoscopy, and categorized as low (0–5 cm) or middle (6–10 cm) rectal cancer.

The three-tier TME grading constructed by Quirke *et al.*^6^ was used by pathologists when examining the rectal specimen, and registered in the SCRCR as: mesorectal plane: ‘intact mesorectum with only minor irregularities of a smooth mesorectal surface, no defect deeper than 5 mm, no coning toward the distal margin of the specimen, smooth circumferential resection margin on slicing’; intramesorectal plane: ‘moderate bulk to the mesorectum, but irregularity of the mesorectal surface, moderate coning of the specimen is allowed, at no site is the muscularis propria visible, with the exception of the insertion of the levator muscles’; muscularis propria plane: ‘little bulk to the mesorectum with defects down onto the muscularis propria and/or a very irregular circumferential resection margin’.

TNM stage was reported according to the seventh edition of the International Union Against Cancer TNM Classification of Malignant Tumors^15^. R1 resection or non-radical resection was defined as tumour growth at the resection surface. Circumferential resection margin (CRM) was measured as the shortest distance between the tumour and the nearest edge of surgically dissected resection plane. CRM ≤1 mm was defined as positive.

### Outcomes of interest

The primary outcomes were cancer recurrence and survival. Recurrences were divided into local and distant recurrences. Local recurrence was defined as an intrapelvic tumour growth (with or without distant recurrence), and distant recurrence as tumour growth outside the pelvis (with or without local recurrence). Overall survival was calculated as time from surgery to death from any cause. Relative survival was defined as the ratio of the observed survival to the expected survival in a general population, matched regarding age, sex and year of surgery. Mortality rate data was retrieved from the Human Mortality Database^16^.

Patients with postoperative death within 30 days, or cancer recurrence discovered within 90 days after surgery, were excluded from recurrence and survival analyses, as a recurrence within this timeframe indicated a disseminated disease at the time of surgery. Patients lacking data regarding recurrence were also excluded from recurrence analyses. Recurrence-free patients with less than 3 years follow-up were censored from the Cox regression recurrence analyses. Date of death was obtained from the National Cause of Death Register at the end of data collection (29 May 2021). Follow-up time was measured from date of surgery to the date of recurrence, death, censoring or data extraction.

The secondary outcome was risk factors for intramesorectal and muscularis propria resection. Age, sex, BMI, tumour level, clinical tumour stage (cT), preoperative oncological treatment, surgical approach (open or minimally invasive), estimated blood loss, duration of surgery and type of surgery (AR, APR or Hartmann’s) were considered variables of interest, and analysed as potential risk factors for intramesorectal and muscularis propria resection.

### Statistical methods

Continuous data are presented as median, with interquartile range (i.q.r.). Categorical data are presented as absolute numbers with percentages. The Kruskal–Wallis test and the chi^2^ test were used when appropriate to test for differences in patient characteristics, pre- and perioperative data and histopathological data between the TME grades. Unadjusted and adjusted Cox regression analyses were performed, relating the TME grades to local recurrence, distant recurrence, overall survival and relative survival, presented as hazard ratios (HR) with 95% confidence intervals (c.i.). A directed acyclic graph was made to identify relevant variables and potential confounders.

The Ederer II method was used for calculations of relative survival. Overall survival and relative survival were calculated with Kaplan–Meier analyses. Differences in survival for the different TME grades were compared using the log-rank test. Logistic multiple regression models were used to test for risk factors for intramesorectal or muscularis propria grade.

IBM SPSS® Statistics for Windows, version 28 and Stata 16.1 (StataCorp, College Station, TX, USA) were used for statistical analyses.

## Results

### Study population

**Fig. 1:**
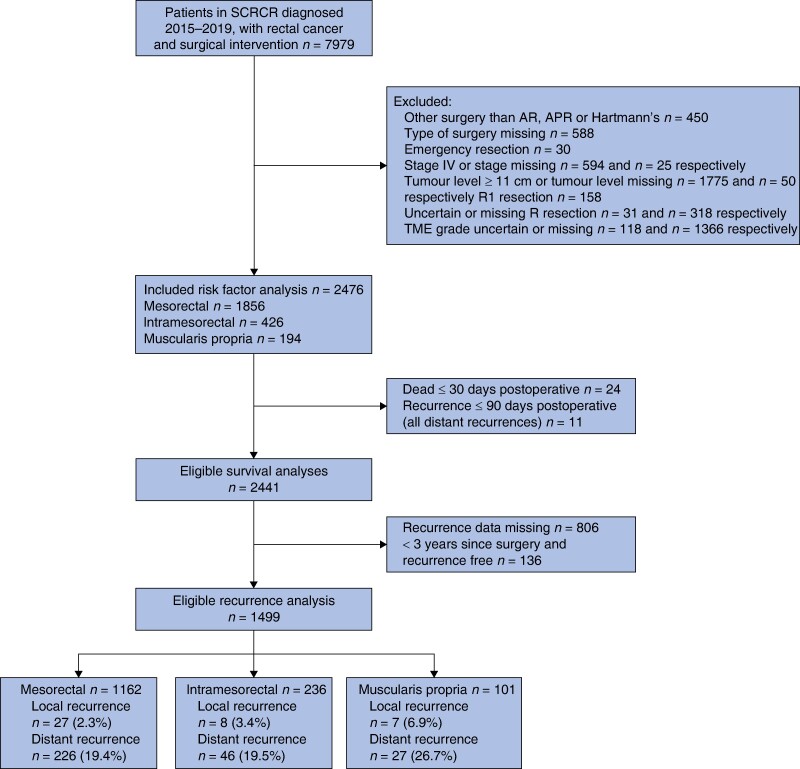
Study flow chart SCRCR, Swedish Colorectal Cancer Registry; AR, anterior resection; APR, abdominoperineal resection; R1, non-radical resection; TME, total mesorectal excision; recurrence 90 days postoperative, all distant recurrences.

In total, data from 7979 patients, diagnosed with rectal cancer between 1 January 2015 and 31 December 2019, who had undergone surgical intervention, were extracted from the SCRCR. After primary exclusions, a total of 2476 patients remained, of which 1856 (75%) were graded mesorectal, 426 (17%) intramesorectal and 194 (8%) muscularis propria (*Fig. 1*).

Twenty-four patients who died within 30 days and 11 patients who developed cancer recurrence within 90 days of surgery were excluded in the Cox regression survival analyses, leaving 2441 patients; 1830 (75%) mesorectal, 422 (17%) intramesorectal and 189 (8%) muscularis propria. Four patients with unknown vital status due to emigration were censored in survival analysis. Median follow-up for survival was 44 (1–77) months in the mesorectal group, 39 (1–77) months in the intramesorectal group and 36 (4–75) months in the muscularis propria group.

In the analysis of recurrences, 136 recurrence-free patients with less than 3 years follow-up and 806 patients lacking data regarding recurrence were excluded, leaving 1499 patients (1162 (78%) mesorectal, 236 (16%) intramesorectal and 101 (7%) muscularis propria (*Fig. 1*)).

In the Cox regression analyses for recurrence, the 136 recurrence-free patients with less than 3 years follow-up time since surgery were instead censored, leaving 1635 patients for analysis.

Patient characteristics, pre- and perioperative data are shown in *Table 1*. Histopathological data are shown in *Table 2*. There was a difference in age, BMI, tumour height, type of surgery, surgical approach, duration of surgery, perioperative blood loss, intraoperative perforation, CRM and perineural growth, when comparing the three TME groups.

**Table 1:** Demographics and pre- and perioperative data of 2476 patients with stage I–III rectal cancer and a tumour level ≤10 cm, who underwent an elective R0 anterior resection, abdominoperineal resection or Hartmann’s procedure

	Mesorectal	Intramesorectal	Muscularis propria	*P*
	(*n* = 1856)	(*n* = 426)	(*n* = 194)	
Age (years), median (i.q.r.)	70 (62–75)	71 (64–75)	72 (64–76)	**0**.**021**
**Sex**				0.057
Female	700 (37.7)	160 (37.6)	90 (46.4)	
Male	1156 (62.3)	266 (62.4)	104 (53.6)	
**BMI (kg/m^2^), median (i.q.r.)**	25.4 (23.1–28.4)	26.1 (23.4–29.1)	26.1 (23.2–28.9)	**0**.**020**
Missing	10 (0.5)	4 (0.9)	3 (1.5)	
**ASA grade**				0.080
I and II	1353 (72.9)	303 (71.1)	130 (67.0)	
III and IV	461 (24.8)	115 (27.0)	63 (32.5)	
Missing	42 (2.3)	8 (1.9)	1 (0.5)	
**Tumour height (cm)**				**<0**.**001**
Low 0–5	629 (33.9)	171 (40.1)	122 (62.9)	
Medium 6–10	1227 (66.1)	255 (59.9)	72 (37.1)	
**Clinical stage**				0.337
I	386 (20.8)	97 (22.8)	51 (26.3)	
II	373 (20.1)	79 (18.5)	35 (18.0)	
III	1056 (56.9)	239 (56.1)	99 (51.0)	
Missing/not assessable	41 (2.2)	11 (2.6)	9 (4.6)	
**cT stage**				0.113
cT1–cT2	536 (28.9)	141 (33.1)	70 (36.1)	
cT3	1018 (54.8)	220 (51.6)	89 (45.9)	
cT4	273 (14.7)	62 (14.6)	28 (14.4)	
cTx	27 (1.5)	3 (0.7)	7 (3.6)	
**Preoperative oncological treatment**				0.085
None	490 (26.4)	103 (24.2)	51 (26.3)	
RT	916 (49.4)	233 (54.7)	107 (55.2)	
CT + RT/CRT	443 (23.9)	86 (20.2)	34 (17.5)	
**Type of surgery**				**<0**.**001**
AR	807 (43.5)	140 (32.9)	35 (18.0)	
APR	814 (43.9)	242 (56.8)	144 (74.2)	
Hartmann’s procedure	235 (12.7)	44 (10.3)	15 (7.7)	
**Surgical approach**				**<0**.**001**
Open	893 (48.1)	161 (37.8)	71 (36.6)	
Minimally invasive‡	962 (51.8)	265 (62.2)	123 (63.4)	
Conversion to open	104 (10.8)	20 (7.5)	9 (7.3)	0.181
**Duration of surgery (min), median (i.q.r.)**	324 (242–415)	370 (282–448)	364 (297–433)	**<0**.**001**
Missing	23 (1.2)	9 (2.1)	3 (1.5)	
**Estimated blood loss (ml), median (i.q.r.)**	175 (50–400)	200 (100–500)	200 (60–470)	**0**.**017**
Missing	30 (1.6)	10 (2.3)	9 (4.6)	
**Intraoperative perforation**				<**0**.**001**
Yes	62 (3.3)	21 (4.9)	29 (14.9)	
No	1782 (96.0)	404 (94.8)	164 (84.5)	

Values are *n* (%) unless stated otherwise. Numbers in bold are statistically significant. ‡Includes laparoscopic (417 of 1350) and robotic (933 of 1350) surgery. BMI, body mass index; ASA, American Society of Anesthesiologists; c, clinical; T, tumour; RT, radiotherapy; CT, chemotherapy; CRT, chemoradiotherapy; AR, anterior resection; APR, abdominoperineal resection; i.q.r., interquartile range. Clinical stage missing includes only M0 with missing T or N, nodal stage. Missing/not assessable data less than 1% not shown in table. (Surgical approach: *n* = 1 mesorectal; preoperative treatment: *n* = 7 mesorectal, *n* = 4 intramesorectal, *n* = 2 muscularis propria; intraoperative perforation: *n* = 12 meso-rectal, *n* = 1 intramesorectal, *n* = 1 muscularis propria.)

**Table 2:** Histopathological data of 2476 patients with stage I–III rectal cancer and a tumour level ≤10 cm, who underwent an elective radical anterior resection, abdominoperineal resection or Hartmann’s procedure

	Mesorectal	Intramesorectal	Muscularis propria	*P*
	(*n* = 1856)	(*n* = 426)	(*n* = 194)	
**Tumour differentiation**				0.377
Low grade	1490 (80.3)	326 (76.5)	160 (82.5)	
High grade	232 (12.5)	61 (14.3)	22 (11.3)	
Missing/not assessable	134 (7.2)	39 (9.2)	12 (6.2)	
**CRM**				**0**.**002**
Negative (>1 mm)	1700 (91.6)	383 (89.9)	172 (88.7)	
Positive (1 mm)	81 (4.4)	26 (6.1)	20 (10.3)	
Missing	75 (4.0)	17 (4.0)	2 (1.0)	
**(y)p stage**				0.953
0–I	672 (36.2)	154 (36.2)	76 (39.2)	
II	517 (27.9)	121 (28.4)	52 (26.8)	
III	661 (35.6)	150 (35.2)	66 (34.0)	
**(y)pT stage**				0.226
0	45 (2.4)	13 (3.1)	1 (0.5)	
1 + 2	814 (43.9)	192 (45.1)	88 (45.4)	
3	913 (49.2)	202 (47.4)	90 (46.4)	
4	84 (4.5)	19 (4.5)	15 (7.7)	
**(y)pN stage**				0.889
0	1189 (64.1)	275 (64.6)	128 (66.0)	
1 + 2	661 (35.6)	150 (35.2)	66 (34.0)	
**Number of lymph nodes retrieved**				0.467
≥12	1655 (89.2)	372 (87.3)	174 (89.7)	
<12	193 (10.4)	53 (12.4)	20 (10.3)	
**Tumour deposits**				0.864
Yes	252 (13.6)	54 (12.7)	27 (13.9)	
No	1586 (85.5)	369 (86.7)	166 (85.6)	
Missing	18 (1.0)	4 (0.9)	1 (0.5)	
**Vascular invasion**				0.179
Yes	442 (23.8)	116 (27.2)	55 (28.4)	
No	1404 (75.6)	309 (72.5)	139 (71.6)	
**Perineural growth**				**0**.**013**
Yes	297 (16.0)	77 (18.1)	47 (24.2)	
No	1550 (83.5)	348 (81.7)	147 (75.8)	

Values are *n* (%). Numbers in bold are statistically significant. CRM, circumferential resection margin; (y), staging after preoperative treatment; p, pathologic; T, tumour; N, nodal. Missing/not assessable data less than 1% not shown in table: ((y)p stage: *n* = 6 mesorectal, *n* = 1 intramesorectal; number of lymph nodes retrieved: *n* = 8 mesorectal, *n* = 1 intramesorectal; vascular invasion: *n* = 10 mesorectal, *n* = 1 intramesorectal; perineural growth: *n* = 9 mesorectal, *n* = 1 intramesorectal).

Adjuvant therapy was given to 317 (21%), 88 (21%) and 34 (18%) in the mesorectal, intramesorectal and muscularis propria groups respectively, with around 20% missing data in all three groups.

Patient characteristics, pre- and perioperative data, histopathological data for 1484 patients with no TME grade (118 (3%) patients with a non-assessable TME grade and 1366 patients (34%) with missing TME grade) are shown in *Supplementary material, Tables S1–S2*. Patients with no TME grade were more frequently operated on with a minimally invasive technique and had more conversions to open surgery. They had a higher proportion of abdominoperineal resections and differed from patients with a TME grade in cT stage and in pathological tumour stage assessed after neoadjuvant oncological treatment ((y)pT) stage and CRM, and more frequently had perineural growth and vascular invasion compared with the patients with a TME grade. Yearly frequencies of the different TME grades are shown in *Supplementary material, Table S3*.

### Recurrence

Local recurrence occurred in 27 of 1162 (2.3%), 8 of 236 (3.4%) and 7 of 101 (6.9%) in the mesorectal, intramesorectal and muscularis propria group respectively. In the adjusted Cox regression analyses, the worst TME grading, muscularis propria resection, was independently associated with a higher local recurrence rate (HR 2.73, 95% c.i. 1.07 to 7.0, *P* = 0.036) (*Table 3*). Of the patients included in the recurrence analysis, 56 patients had an intraoperative perforation, but only two of these patients developed local recurrence: one in the intramesorectal group and one in the muscularis propria group. The perforation was described as tumour-close (based on the individual surgeon’s estimation) in 26 of the 56 cases. None of these 26 patients developed a local recurrence during the study interval.

**Table 3:** **Unadjusted and adjusted** *  **Cox regression relating TME grade to local recurrence, distant recurrence, overall survival and relative survival**

	Local recurrence		Distant recurrence		Overall survival		Relative survival	
TME grade	HR (95% c.i.)	*P*	HR (95% c.i.)	*P*	HR (95% c.i.)	*P*	HR (95% c.i.)	*P*
	**Unadjusted**							
	*n* = 1635		*n* = 1635		*n* = 2441			
Mesorectal	1.00		1.00		1.00		1.00	
Intramesorectal	1.91 (0.84, 4.30)	0.121	0.90 (0.61, 1.31)	0.567	0.92 (0.69, 1.23)	0.577	0.84 (0.45, 1.55)	0.570
Muscularis propria	2.82 (1.14, 6.99)	**0.025**	1.08 (0.65, 1.77)	0.772	1.10 (0.74, 1.64)	0.632	1.34 (0.65, 2.74)	0.427
	**Adjusted** *							
	*n* = 1620		*n* = 1620		*n* = 2420			
Mesorectal	1.00		1.00		1.00		1.00	
Intramesorectal	2.03 (0.89, 4.61)	0.090	0.89 (0.61, 1.30)	0.540	0.91 (0.68, 1.21)	0.506	0.95 (0.53, 1.69)	0.851
Muscularis propria	3.24 (1.25, 8.39)	**0.015**	1.00 (0.60, 1.67)	0.996	1.01 (0.67, 1.51)	0.965	1.40 (0.70, 2.80)	0.338

*Adjusted for age, sex, body mass index, clinical T stage, surgical approach (open or minimally invasive), tumour level and preoperative oncological treatment. Numbers in bold are statistically significant. TME, total mesorectal excision.

Perineural involvement was higher in the muscularis propria group, as reported in *Table 2*. If excluding all patients with perineural growth from the 1499 patients eligible for recurrence analysis, the local recurrence rate was 17 of 989 = 1.7% in the mesorectal group, 4 of 197 = 2% in the intramesorectal group and 3 of 77 = 3.9% in the muscularis propria group.

Distant recurrence occurred in 226 of 1162 (19.4%), 46 of 236 (19.5%) and 27 of 101 (26.7%) of the patients in the mesorectal, intramesorectal and muscularis propria group respectively, with no statistical differences (*Table 3*).

Local and distant recurrence rate at 3 years for patients with no TME grade is shown in *Supplementary material*, *Table S4*.

An additional Cox regression analysis was performed, including the patients with no TME grade. No TME grade was not associated with a higher risk of local or distant recurrence (*Supplementary material*, *Table S5*).

The directed acyclic graph made for choosing relevant variables is shown in *Supplementary material, Fig. S1*.

The minimal sufficient adjustments for estimating the total effect of TME grade on survival were BMI, cT stage, sex, surgical approach, tumour level and type of surgery. In addition, age and preoperative oncological treatment were added to the model. As the decision of type of surgery in the majority of cases is based on the tumour level, this variable was considered to be a collinear variable and was omitted from Cox regression analyses.

### Survival

**Fig. 2:**
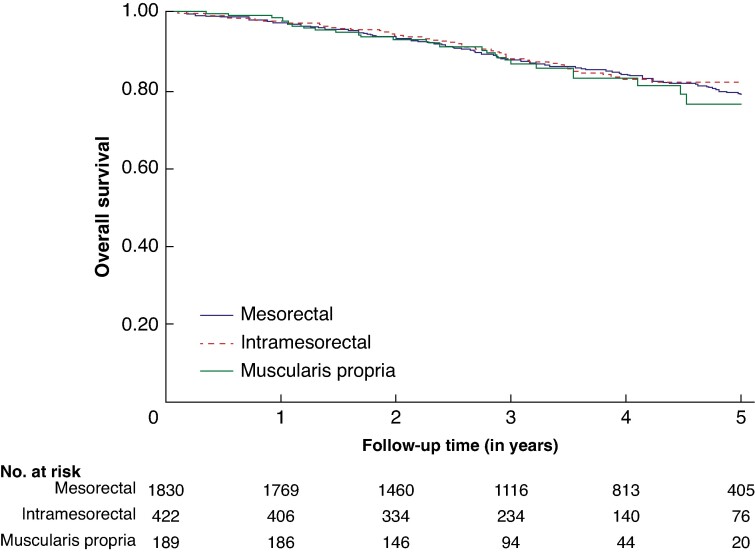
Overall survival in patients with mesorectal, intramesorectal and muscularis propria resection

Kaplan–Meier curves for overall survival and relative survival are shown respectively in *Fig. 2*, *Fig. S2* (*Supplementary material*).There was no difference in overall survival or relative survival between the three TME groups (*Table 3*). Overall survival rate at 3 years for patients with no TME grade is shown in *Supplementary material*, *Table S3*. An additional Cox regression analysis including the patients without a TME grade showed no association between no TME grade and worse overall survival or relative survival (*Supplementary material*, *Table S5*).

### Risk factors for intramesorectal and muscularis propria grade

In multivariable analyses, APR and minimally invasive surgery were risk factors for both intramesorectal and muscularis propria resection. Female sex, tumour level < 5 cm, blood loss > 800 ml and intraoperative perforation were risk factors for muscularis propria but not for intramesorectal resection. Duration of surgery > 9 h was a risk factor for intramesorectal but not for muscularis propria resection (*Table 4*).

**Table 4:** Risk factors for intramesorectal or muscularis propria grade, results from logistic multiple regression models

	Intramesorectal		Muscularis propria	
	OR (95% c.i.)	*P*	OR (95% c.i.)	*P*
**Age (years)**				
≤75	1.00 (Ref.)		1.00 (Ref.)	
>75	1.09 (0.83, 1.42)	0.539	1.11 (0.77, 1.61)	0.568
**Sex**				
Male	1.00 (Ref.)		1.00 (Ref.)	
Female	1.13 (0.90, 1.42)	0.302	1.61 (1.16, 2.23)	0.004
**BMI (kg/m^2^)**				
<18.5	0.67 (0.26, 1.72)	0.402	1.07 (0.36, 3.17)	0.908
18.5–29.9	1.00 (Ref.)		1.00 (Ref.)	
≥30.0	1.32 (1.00, 1.73)	0.050	1.27 (0.85, 1.90)	0.249
**Tumour height (cm)**				
Low, 0–5	0.90 (0.67, 1.21)	0.488	2.02 (1.32, 3.09)	**0**.**001**
Medium, 6–10	1.00 (Ref.)		1.00 (Ref.)	
**cT stage**				
cT1–cT2	1.00 (Ref.)		1.00 (Ref.)	
cT3	0.83 (0.64, 1.09)	0.189	0.89 (0.61, 1.32)	0.573
cT4	0.86 (0.58, 1.28)	0.456	0.78 (0.45, 1.38)	0.403
x	0.40 (0.12, 1.37)	0.146	1.78 (0.71, 4.48)	0.221
**Preoperative oncological treatment**				
None	1.00 (Ref.)		1.00 (Ref.)	
RT	1.16 (0.87, 1.56)	0.313	0.84 (0.56, 1.28)	0.426
CT + RT/CRT	0.92 (0.63, 1.36)	0.690	0.59 (0.33, 1.05)	0.072
**Type of surgery**				
AR	1.00 (Ref.)		1.00 (Ref.)	
APR	1.66 (1.21, 2.26)	**0.002**	2.37 (1.42, 3.96)	**0.001**
Hartmann’s procedure	1.20 (0.81, 1.77)	0.365	1.47 (0.75, 2.85)	0.260
**Surgical approach**				
Open	1.00 (Ref.)		1.00 (Ref.)	
Minimally invasive	1.65 (1.27, 2.13)	**<0**.**001**	1.72 (1.18, 2.49)	**0**.**004**
**Duration of surgery (h)**				
<5	1.00 (Ref.)		1.00 (Ref.)	
5–9	1.21 (0.93, 1.57)	0.153	1.18 (0.80, 1.73)	0.395
>9	1.70 (1.07, 2.69)	**0**.**024**	1.37 (0.70, 2.68)	0.354
**Perioperative blood loss (ml)**				
<200	1.00 (Ref.)		1.00 (Ref.)	
200–800	1.33 (1.04, 1.71)	**0**.**025**	1.24 (0.86, 1.81)	0.249
>800	1.42 (0.96, 2.11)	0.081	1.98 (1.17, 3.33)	**0**.**010**
**Intraoperative perforation**				
No	1.00 (Ref.)		1.00 (Ref.)	
Yes	1.22 (0.72, 2.05)	0.457	3.92 (2.37, 6.48)	**<0**.**001**

Numbers in bold are statistically significant. OR, odds ratio; c.i., confidence interval; BMI, body mass index; c, clinical; RT, radiotherapy; CT, chemotherapy; CRT, chemoradiotherapy; AR, anterior resection; APR, abdominoperineal resection.

## Discussion

In this national population-based cohort study, patients with TME grade muscularis propria had a higher risk of developing local recurrence, but the TME grade had no impact on distant recurrence, overall survival or relative survival. The association between TME grade and local recurrence rate is in line with several earlier studies^6–11^. While there are studies showing no correlation between local recurrence and TME grade, these are small or have a relatively short follow-up^5,13,17,18^. The lack of correlation between distant recurrence or overall survival and TME grade is consistent with two recent studies^10,11^; one of them, however, found mesorectal resections to correlate with increased 5 year disease-free survival^11^. Muscularis propria grade has been shown to predict distant recurrence, disease-free survival and overall survival with a two-tier grading, combining mesorectal and intramesorectal *versus* muscularis propria^13^. Two-tier grades, combining intramesorectal with either mesorectal or muscularis propria grade, were used in some earlier studies. In this population-based study the number of patients was considered sufficient for analysing the three grades separately. Several factors were associated with intramesorectal or muscularis propria grade dissection. Low tumour height (< 5 cm) was associated with muscularis propria resection, and APR, the procedure of choice in low tumours, was related to both intramesorectal and muscularis propria grade. The perineal part of an APR is challenging, since no well-defined dissection plane is offered, and a higher incidence of intramesorectal and muscularis propria grade has been reported^5–8,10,12,17,19^. A higher rate of CRM involvement, perforation, local recurrence and poorer overall survival have been described in APR compared with AR^20,21^.

Minimally invasive surgery was associated with intramesorectal and muscularis propria grade. Two meta-analyses and two randomized controlled trials have reported a lower incidence of complete TME resections with minimally invasive surgery^22–25^, whereas some studies have found no association between TME quality and surgical approach^19,26,27^. A recent study comparing robotic and open mesorectal excision reported similar oncologic outcomes^28^. In 2015, robotic surgery was being introduced in many parts of Sweden, and the finding of more intramesorectal or muscularis propria resections could be related to the learning curve. The increasing number of intramesorectal and muscularis propria grading during the study interval is perhaps due to a more careful examination by pathologists, as the awareness of TME grade increased from 2015, when introduced.

The difficulties of rectal cancer surgery in men are often stressed, whereas rectal surgery in females is generally considered easier, partly due to their wider pelvis. In this study, however, female sex was a risk factor for muscularis propria resection, a finding supported by one previous study^17^. The reason for this finding is unclear, but may reflect higher attentiveness during the mesorectal dissection in men, since it is considered more difficult.

A long duration of surgery, intraoperative perforation and high blood loss were all associated with intramesorectal and/or muscularis propria grade. All these risk factors probably indicate an advanced tumour, or strenuous surgery, with a following increased risk of not achieving mesorectal resection.

This study has a few limitations. Thirty-seven per cent of the cases in this study had no TME grade reported by the local pathologists. While this weakens the study, no TME grade was not associated with a higher local or distant recurrence rate or worse overall or relative survival, thus the reason for not grading did not seem to correlate with difficulty grading an inferior specimen. Fewer missing TME grades would have been a strength but since the study cohort was large and there were no obvious signs of selection bias, the results are considered reliable. Also, since this was a retrospective register-based study, no validation of the pathologists’ TME grade could be performed. Thirty per cent of the patients had no registered 3 year follow-up, mainly due to shorter follow-up at SCRCR data extraction. In Sweden, rectal cancer patients routinely undergo computer tomography of the chest and abdomen, and outpatient visit (including clinical examination and rigid sigmoidoscopy when possible), at 1 and 3 years postresection surgery. The SCRCR registers events at the 3 year and 5 year follow-up. Recurrences discovered earlier can be registered separately, but are frequently registered *a posteriori* in conjunction with the 3 year or 5 year follow-up. Therefore, this study could underreport recurrences. The follow-up time could also have been too short to detect any differences in distant recurrence or survival. The proportion of patients with an intraoperative tumour perforation was higher in the muscularis propria group. However, none of the patients with a tumour-close perforation developed local recurrence in this study, even though perforation is an established risk factor for local recurrence.

While perineural growth was higher in the muscularis propria group, when excluding patients with perineural growth from analyses, the local recurrence rate was still higher in the muscularis propria group, indicating that muscularis propria grade is an independent risk factor for local recurrence. In addition, multivariable analysis of local recurrence was performed despite few events (8 variables and 43 events), as the rule of 10 events per variable might be too strict and five events per variable may give equal results^29^.

The findings reported here stress the importance of good surgical quality and underline TME grade as a measurement of that. Tumour level, female sex, blood loss and intraoperative perforation were risk factors for muscularis propria resection, and APR and minimally invasive surgery were risk factors for both intramesorectal and muscularis propria resection. Extra caution is warranted in these patients.

## Supplementary Material

zrae071_Supplementary_Data

## Data Availability

Data are available through the SCRCR, however, restrictions apply to the availability, and are therefore not publicly available.

## References

[1] Heald RJ, Ryall RD. Recurrence and survival after total mesorectal excision for rectal cancer. Lancet 1986;1:1479–1482 2425199 10.1016/s0140-6736(86)91510-2

[2] Peeters KC, Marijnen CA, Nagtegaal ID, Kranenbarg EK, Putter H, Wiggers T et al The TME trial after a median follow-up of 6 years: increased local control but no survival benefit in irradiated patients with resectable rectal carcinoma. Ann Surg 2007;246:693–701 17968156 10.1097/01.sla.0000257358.56863.ce

[3] Martling AL, Holm T, Rutqvist LE, Moran BJ, Heald RJ, Cedemark B. Effect of a surgical training programme on outcome of rectal cancer in the County of Stockholm. Stockholm Colorectal Cancer Study Group, Basingstoke Bowel Cancer Research Project. Lancet 2000;356:93–96 10963244 10.1016/s0140-6736(00)02469-7

[4] Heald RJ, Husband EM, Ryall RD. The mesorectum in rectal cancer surgery–the clue to pelvic recurrence? Br J Surg 1982;69:613–616 6751457 10.1002/bjs.1800691019

[5] Nagtegaal ID, van de Velde CJ, van der Worp E, Kapiteijn E, Quirke P, van Krieken JH et al Macroscopic evaluation of rectal cancer resection specimen: clinical significance of the pathologist in quality control. J Clin Oncol 2002;20:1729–1734 11919228 10.1200/JCO.2002.07.010

[6] Quirke P, Steele R, Monson J, Grieve R, Khanna S, Couture J et al Effect of the plane of surgery achieved on local recurrence in patients with operable rectal cancer: a prospective study using data from the MRC CR07 and NCIC-CTG CO16 randomised clinical trial. Lancet 2009;373:821–828 19269520 10.1016/S0140-6736(09)60485-2 PMC2668948

[7] Maslekar S, Sharma A, Macdonald A, Gunn J, Monson JR, Hartley JE. Mesorectal grades predict recurrences after curative resection for rectal cancer. Dis Colon Rectum 2007;50:168–175 17160574 10.1007/s10350-006-0756-2

[8] Leite JS, Martins SC, Oliveira J, Cunha MF, Castro-Sousa F. Clinical significance of macroscopic completeness of mesorectal resection in rectal cancer. Colorectal Dis 2011;13:381–386 20002696 10.1111/j.1463-1318.2009.02153.x

[9] Bosch SL, Nagtegaal ID. The importance of the pathologist’s role in assessment of the quality of the mesorectum. Curr Colorectal Cancer Rep 2012;8:90–98 22611342 10.1007/s11888-012-0124-7 PMC3343235

[10] Kitz J, Fokas E, Beissbarth T, Strobel P, Wittekind C, Hartmann A et al Association of plane of total mesorectal excision with prognosis of rectal cancer: secondary analysis of the CAO/ARO/AIO-04 phase 3 randomized clinical trial. JAMA Surg 2018;153:e181607 29874375 10.1001/jamasurg.2018.1607 PMC6142959

[11] Kim JC, Park SH, Kim J, Kim CW, Park IJ, Yoon YS et al Involvement of tissue changes induced by neoadjuvant treatment in total mesorectal excision (TME): novel suggestions for determining TME quality. Int J Colorectal Dis 2022;37:1289–1300 35513539 10.1007/s00384-022-04165-z

[12] Garcia-Granero E, Faiz O, Munoz E, Flor B, Navarro S, Faus C et al Macroscopic assessment of mesorectal excision in rectal cancer: a useful tool for improving quality control in a multidisciplinary team. Cancer 2009;115:3400–3411 19479978 10.1002/cncr.24387

[13] Leonard D, Penninckx F, Laenen A, Kartheuser A; Procare. Scoring the quality of total mesorectal excision for the prediction of cancer-specific outcome. Colorectal Dis 2015;17:O115–O122 25714054 10.1111/codi.12931

[14] Moberger P, Skoldberg F, Birgisson H. Evaluation of the Swedish Colorectal Cancer Registry: an overview of completeness, timeliness, comparability and validity. Acta Oncol 2018;57:1611–1621 30477372 10.1080/0284186X.2018.1529425

[15] Sobin LH, Sobin LH, Gospodarowicz MK, Wittekind C; International Union against Cancer. TNM classification of Malignant Tumours. 7th ed. Chichester, UK; Hoboken, NJ: Wiley-Blackwell, 2009

[16] Human Mortality Database. Max Planck Institute for Demographic Research (Germany), University of California, Berkeley (USA), and French Institute for Demographic Studies (France). https://www.mortality.org (accessed 28 November 2023)

[17] Jeyarajah S, Sutton CD, Miller AS, Hemingway D; Leicester Colorectal Specialist Group. Factors that influence the adequacy of total mesorectal excision for rectal cancer. Colorectal Dis 2007;9:808–815 17441969 10.1111/j.1463-1318.2007.01256.x

[18] Garoufalia Z, Freund MR, Gefen R, Meyer R, DaSilva G, Weiss EG et al Does completeness of the mesorectal excision still correlate with local recurrence? Dis Colon Rectum 2023;66:898–904 36649177 10.1097/DCR.0000000000002551

[19] Sapci I, Velazco JS, Xhaja X, Aiello A, Gorgun E, Stocchi L et al Factors associated with noncomplete mesorectal excision following surgery for rectal adenocarcinoma. Am J Surg 2019;217:465–468 30454839 10.1016/j.amjsurg.2018.10.051

[20] Marr R, Birbeck K, Garvican J, Macklin CP, Tiffin NJ, Parsons WJ et al The modern abdominoperineal excision: the next challenge after total mesorectal excision. Ann Surg 2005;242:74–82 15973104 10.1097/01.sla.0000167926.60908.15 PMC1357707

[21] Nagtegaal ID, van de Velde CJ, Marijnen CA, van Krieken JH, Quirke P; Dutch Colorectal Cancer Group et al Low rectal cancer: a call for a change of approach in abdominoperineal resection. J Clin Oncol 2005;23:9257–9264 16361623 10.1200/JCO.2005.02.9231

[22] Creavin B, Kelly ME, Ryan E, Winter DC. Meta-analysis of the impact of surgical approach on the grade of mesorectal excision in rectal cancer. Br J Surg 2017;104:1609–1619 29044484 10.1002/bjs.10664

[23] Stevenson AR, Solomon MJ, Lumley JW, Hewett P, Clouston AD, Gebski VJ et al Effect of laparoscopic-assisted resection vs open resection on pathological outcomes in rectal cancer: the ALaCaRT randomized clinical trial. JAMA 2015;314:1356–1363 26441180 10.1001/jama.2015.12009

[24] Fleshman J, Branda M, Sargent DJ, Boller AM, George V, Abbas M et al Effect of laparoscopic-assisted resection vs open resection of stage II or III rectal cancer on pathologic outcomes: the ACOSOG Z6051 randomized clinical trial. JAMA 2015;314:1346–1355 26441179 10.1001/jama.2015.10529 PMC5140087

[25] Martinez-Perez A, Carra MC, Brunetti F, de'Angelis N. Pathologic outcomes of laparoscopic vs open mesorectal excision for rectal cancer: a systematic review and meta-analysis. JAMA Surg 2017;152:e165665 28196217 10.1001/jamasurg.2016.5665

[26] Silva-Velazco J, Stocchi L, Valente MA, Church JM, Liska D, Gorgun E et al The relationship between mesorectal grading and oncological outcome in rectal adenocarcinoma. Colorectal Dis 2019;21:315–325 30565830 10.1111/codi.14535

[27] Bonjer HJ, Deijen CL, Abis GA, Cuesta MA, van der Pas MH, de Lange-de Klerk ES et al A randomized trial of laparoscopic versus open surgery for rectal cancer. N Engl J Med 2015;372:1324–1332 25830422 10.1056/NEJMoa1414882

[28] Jimenez-Rodriguez RM, Flynn J, Patil S, Widmar M, Quezada-Diaz F, Lynn P et al Comparing outcomes of robotic versus open mesorectal excision for rectal cancer. BJS Open 2021;5:zrab135 35040943 10.1093/bjsopen/zrab135 PMC8765333

[29] Vittinghoff E, McCulloch CE. Relaxing the rule of ten events per variable in logistic and Cox regression. Am J Epidemiol 2007;165:710–718 17182981 10.1093/aje/kwk052

