# Supplementary material for: Total mesorectal excision quality in rectal cancer surgery affects local recurrence rate but not distant recurrence and survival: population-based cohort study
Source: BJS Open. 2024 Aug 8;8(4):zrae071. doi: 10.1093/bjsopen/zrae071 (PMC11306320; doi:10.1093/bjsopen/zrae071)
Supplement: zrae071_Supplementary_Data [file zrae071_supplementary_data.docx]

**TME quality in rectal cancer surgery affects local recurrence rate but not distant recurrence and survival: a population-based cohort study.**

Åsa Collin^1^, Cecilia Dahlbäck^2,3^, Joakim Folkesson^1^, Pamela Buchwald^2,3^

^1^Department of Surgical Sciences, Uppsala University Hospital, Uppsala, Sweden

^2^ Department of Clinical Sciences Malmö, Lund University, Malmö, Sweden

^3^ Department of Surgery, Skåne University Hospital, Malmö, Sweden

**Corresponding author:** Åsa Collin, Department of Surgical Sciences, Colorectal Surgery, Uppsala University, 751 85 Uppsala, Sweden (e-mail: asa.collin@uu.se)

**Supplementary Materials - Index**

| **Supplementary Methods** | *page 2* |
| --- | --- |
| **Supplementary Results** | *page 3* |
| **Supplementary Figures and Tables** |  |
| Table S1. Demographics and pre- and perioperative data for patients with no TME grade | *page 4* |
| Table S2. Histopathological data for patients with no TME grade  Table S3. Proportion of the different TME grades per year of surgery  Table S4. Recurrence and survival rate at three years for patients with no TME grade  Table S5. Cox regression analysis for recurrence and survival including no TME grade  Figure S1. Directed acyclic graph  Figure S2. Relative survival (mesorectal, intramesorectal and muscularis propria grade) | *page 5*  *page 6*  *page 7*  *page 8*  *page 9*  *page 10* |

**Supplementary Methods**

Data was recorded for patients with stage I-III rectal cancer and a tumor level ≤10 cm who underwent an elective anterior resection, abdominoperineal resection, or Hartmann’s procedure. Patient characteristics, pre- and perioperative data and histopathological data were recorded for patients with a registered TME grade and patients without a registered TME grade (TME grade missing or not assessable included).

Recurrence and survival rate at three years were analyzed in patients with TME grade mesorectal, intramesorectal, muscularis propria and patients with no registered TME grade. Patients with post-operative mortality within 30 days, or cancer recurrence discovered within 90 days postoperatively were excluded from survival and recurrence analyses. Patients lacking data regarding recurrence were excluded in recurrence analysis and recurrence-free patients with less than three years follow-up were censored in the Cox regression analysis.

Survival for mesorectal, intramesorectal and muscularis propria group was calculated with Kaplan-Meier analysis. Differences in survival for the different TME grades were compared using the log-rank test.

**Supplementary Results**

In all, a total of 3960 patients with stage I-III rectal cancer and a tumor level ≤10 cm who underwent an elective R0 anterior resection, abdominoperineal resection, or Hartmann’s procedure were included. Of these, 1484 patients had no registered TME grade (1336 patients were missing a TME grade and 118 patients had been registered as “TME grade not assessable”). Patients with no TME grade (TME grade missing or not assessable) were more frequently operated with minimally invasive technique and had more conversions to open surgery. They had a higher proportion of abdominoperineal resections and differed from patients with a TME grade in cT stage and (y)pT stage and CRM, and more frequently had perineural growth and vascular invasion compared to the patients with a TME grade (Table S1-S2).

The proportion of patients with TME grade missing increased from 32.3% in 2015 to 41% in 2020 (Table S3). Three-year local recurrence and distant recurrence rate and overall survival in the mesorectal, intramesorectal, muscularis propria and no TME grade group respectively is seen in Table S4.

No registered TME grade was not associated with a higher risk of local recurrence or distant recurrence or worse overall survival or relative survival in the Cox regression analysis (Table S5).

The directed acyclic graph made to identify relevant variables (age, sex, BMI, tumour level, clinical tumour stage (cT), preoperative oncological treatment) and potential confounders is seen in Figure S1.

Log rank test showed no difference in overall or relative survival between mesorectal, intramesorectal or muscularis propria grade (Fig S2).

**Supplementary Figures and Tables**

**Table S1.** Demographics and pre- and perioperative data of 3 960 patients with stage I-III rectal cancer and a tumour level ≤10 cm who underwent an elective R0 anterior resection, abdominoperineal resection, or Hartmann’s procedure. Patients with and without a registered TME grade.

|  | All patients | TME grade | No TME grade | p |
| --- | --- | --- | --- | --- |
|  | (n=3 960) | (n=2476) | (n=1 484) |  |
| Age (years)* | 70 (62-76) | 70 (62-75) | 70 (62-76) | 0.094 |
| Sex |  |  |  | 0.661 |
| Female | 1 509 (38.1) | 950 (38.4) | 559 (37.7) |  |
| Male | 2 451 (61.9) | 1526 (61.6) | 925 (62.3) |  |
| BMI (kg/m2)* | 25.5 (23.1-28.5) |  | 25.1 (23.1-28.4) | 0.452 |
| ASA grade |  |  |  | 0.338 |
| 1 and 2 | 2 832 (71.5) | 1786 (72.1) | 1 046 (70.5) |  |
| 3 and 4 | 1 041 (26.3) | 639 (25.8) | 402 (27.1) |  |
| Missing | 87 (2.2) | 51 (2.1) | 36 (2.4) |  |
| Tumour height (cm) |  |  |  | 0.055 |
| Low 0-5 | 1 520 (38.4) | 922 (37.2) | 598 (40.3) |  |
| Medium 6-10 | 2 440 (61.6) | 1554 (62.8) | 886 (59.7) |  |
| C Stage |  |  |  | 0.909 |
| I | 864 (21.8) | 534 (21.6) | 330 (22.2) |  |
| II | 780 (19.7) | 487 (19.7) | 293 (19.7) |  |
| III | 2 225 (56.2) | 1394 (56.3) | 831 (56.0) |  |
| Missing/not assessable | 91 (2.3) | 61 (2.5) | 30 (2.0) |  |
| cT stage |  |  |  | 0.029 |
| 1 and 2 | 1 202 (30.4) | 747 (30.2) | 455 (30.7) |  |
| 3 | 2 070 (52.3) | 1327 (53.6) | 743 (50.1) |  |
| 4 | 623 (15.7) | 363 (14.7) | 260 (17.5) |  |
| x | 61 (1.5) | 37 (1.5) | 24 (1.6) |  |
| Preoperative oncological treatment |  |  |  | 0.144 |
| None | 1 080 (27.3) | 644 (26.0) | 436 (29.4) |  |
| RT | 1 979 (50.0) | 1256 (50.7) | 723 (48.7) |  |
| CT + RT /CRT | 880 (22.2) | 563 (22.7) | 317 (21.4) |  |
| Type of surgery |  |  |  | 0.005 |
| AR | 1 495 (37.8) | 982 (39.7) | 513 (34.6) |  |
| APR | 1 987 (50.2) | 1200 (48.5) | 787 (53.0) |  |
| Hartmann's procedure | 478 (12.1) | 294 (11.9) | 497 (33.5) |  |
| Surgical approach |  |  |  | <0.001 |
| Open | 1 709 (43.2) | 1125 (45.4) | 584 (39.4) |  |
| Minimally invasive | 2 248 (56.8) | 1350 (54.5) | 898 (60.5) |  |
| Conversion to open | 271 (12.1) | 133 (9.9) | 138 (15.4) | <0.001 |
| Duration of surgery (min)* | 335 (252-421) | 338 (253-422) | 332 (250-418) | 0.528 |
| Missing | 32 (0.8) | 10 (0.4) | 16 (1.1) |  |
| Estimated blood loss (ml)* | 200 (50-400) | 200 (50-400) | 200 (50-400) | 0.915 |
| Missing | 80 (2.0) | 48 (1.9) | 31 (2.1) |  |
| Intraoperative perforation |  |  |  |  |
| Yes | 170 (4.3) | 112 (4.5) | 58 (3.9) | 0.359 |
| No | 3 766 (95.1) | 2350 (94.9) | 1 416 (95.4) |  |

Values are n (%) unless stated otherwise. * Values are median (i.q.r.). No TME grade includes TME grade missing and TME grade not assessable. BMI, Body Mass Index; ASA, American Society of Anesthesiologists; RT, radiotherapy; CRT, chemoradiotherapy; AR, Anterior resection; APR, Abdomino perineal resection; i.q.r., inter quartile range. CT, Chemotherapy (n = 10 TME grade, n = 6 no TME grade) not shown in table. Missing/not assessable data less than 1% not shown in table: (BMI: n = 17 TME grade, n = 12 no TME grade; cT stage: n = 2 TME grade, n = 2 no TME grade; Preoperative treatment: n = 3 TME grade, n = 2 no TME grade; Surgical approach: n = 1 TME grade, n = 2 no TME grade; Conversion to open: n = 1 TME grade, n = 2 no TME grade; Intraoperative perforation: n = 14 TME grade, n = 10 no TME grade).

**Table S2.** Histopathological data of 3 960 patients with stage I-III rectal cancer and a tumour level ≤10 cm who underwent an elective radical anterior resection, abdominoperineal resection, or Hartmann’s procedure. Patients with and without a registered TME grade.

|  | All patients | TME grade | No TME grade | p |
| --- | --- | --- | --- | --- |
|  | (n=3 960) | (n=2476) | (n=1 484) |  |
| Tumour differentiation |  |  |  | 0.532 |
| Low grade | 3 164 (79.9) | 1976 (79.8) | 1 188 (80.1) |  |
| High grade | 513 (13.0) | 315 (12.7) | 198 (13.3) |  |
| Missing/not assessable | 283 (7.1) | 185 (0.07) | 98 (6.6) |  |
| CRM |  |  |  | 0.024 |
| Negative >1mm | 3 572 (90.2) | 2255 (91.1) | 1 317 (88.7) |  |
| Positive* | 212 (5.4) | 127 (5.1) | 85 (5.7) |  |
| Missing/not assessable | 176 (4.4) | 94 (3.8) | 82 (5.5) |  |
| (y)p Stage |  |  |  | 0.202 |
| 0-I | 1 474 (37.2) | 902 (36.4) | 572 (38.5) |  |
| II | 1 067 (26.9) | 690 (27.9) | 377 (25.4) |  |
| III | 1 403 (35.4) | 877 (35.4) | 526 (35.4) |  |
| (y)pT Stage |  |  |  | 0.002 |
| 0 | 83 (2.1) | 59 (2.4) | 24 (1.6) |  |
| 1+2 | 1 800 (45.5) | 1094 (44.2) | 706 (47.6) |  |
| 3 | 1 859 (46.9) | 1205 (48.7) | 654 (44.1) |  |
| 4 | 215 (5.4) | 118 (4.8) | 97 (6.5) |  |
| (y)pN stage |  |  |  | 0.953 |
| 0 | 2 543 (64.2) | 1592 (64.3) | 951 (64.1) |  |
| 1+2 | 1403 (35.4) | 877 (35.4) | 526 (35.4) |  |
| Number of lymph nodes retrieved |  |  |  | 0.147 |
| ≥12 | 3 492 (88.2) | 2201 (88.9) | 1 291 (87.0) |  |
| <12 | 447 (11.3) | 266 (10.7) | 181 (12.2) |  |
| Tumour deposits |  |  |  | 0.584 |
| Yes | 537 (13.6) | 333 (13.4) | 204 (13.7) |  |
| No | 3 354 (84.7) | 2121 (85.7) | 1 233 (83.1) |  |
| Missing | 69 (1.7) | 22 (0.9) | 47 (3.2) |  |
| Vascular invasion |  |  |  |  |
| Yes | 1 032 (26.1) | 613 (24.8) | 419 (28.2) | 0.007 |
| No | 2 888 (72.9) | 1852 (74.8) | 1 036 (69.8) |  |
| Missing | 40 (1.0) | 11 (0.4) | 29 (2.0) |  |
| Perineural growth |  |  |  | <0.001 |
| Yes | 742 (18.7) | 421 (17.0) | 321 (21.6) |  |
| No | 3 184 (80.4) | 2045 (82.6) | 1 139 (76.8) |  |
| Missing | 34 (0.9) | 10 (0.4) | 24 (1.6) |  |

Values are n (%). No TME grade includes TME grade missing and TME grade not assessable. CRM, circumferential resection margin * positive CRM is still R0, as R1 resections were excluded; (y), staging after preoperative treatment; p, pathologic. Missing/not assessable data less than 1% not shown in table: ((y)p Stage: n = 7 TME grade, n = 9 no TME grade; (y)pT stage n = 3 no TME grade; (y)pN stage: n =7 TME grade, n = 7 no TME grade; Number of lymph nodes retrieved: n = 9 TME grade, n = 12 no TME grade).

**Table S3.** Proportion of TME grading shown per year of surgery in patients with stage I-III rectal cancer and a tumour level ≤10 cm who underwent an elective radical anterior resection, abdominoperineal resection, or Hartmann’s procedure.

|  | **Mesorectal** | **Intramesorectal** | **Muscularis propria** | **Not assessable** | **Missing** |
| --- | --- | --- | --- | --- | --- |
| **2015** | 334/650 (51.4) | 58/650 (8.9) | 20/650 (3.1) | 28/650 (4.3) | 210/650 (32.3) |
| **2016** | 505/910 (55.5) | 82/910 (9.0) | 32/910 (3.5) | 42/910 (4.6) | 249/910 (27.4) |
| **2017** | 326/754 (43.2) | 90/754 (11.9) | 43/754 (5.7) | 22/754 (2.9) | 273/754 (36.2) |
| **2018** | 314/694 (45.2) | 85/694 (12.2) | 54/694 (7.8) | 7/694 (1.0) | 234/694 (33.7) |
| **2019** | 293/735 (39.9) | 88/735 (12.0) | 33/735 (4.5) | 10/735 (1.4) | 311/735 (42.3) |
| **2020** | 81/212 (38.2) | 23/212 (10.8) | 12/212 (5.7) | 9/212 (4.2) | 87/212 (41.0) |
|  |  |  |  |  |  |

Values are n (%). Patients diagnosed in 2019, who underwent surgery in 2021 not shown due to few patients (n = 3).

**Table S4.** Recurrence and survival rate at 3 years in patients with stage I-III rectal cancer and a tumour level ≤10 cm who underwent an elective radical anterior resection, abdominoperineal resection, or Hartmann’s procedure with no post-operative mortality within 30 days, or cancer recurrence discovered within 90 days postoperatively. Excluded in recurrence analysis: recurrence-free patients with less than three years follow-up, and patients lacking data regarding recurrence.

|  | Mesorectal | Intramesorectal | Muscularis Propria | No TME grade |
| --- | --- | --- | --- | --- |
| Local recurrence | 27/1 162 (2.3) | 8/236 (3.4) | 7/101 (6.9) | 31/896 (3.5) |
| Distant recurrence | 226/1 162 (19.4) | 46/236 (19.5) | 27/101 (26.7) | 174/896 (19.4) |
| Overall survival | 1 023/1 312 (78.0) | 222/276 (80.4) | 86/114 (75.4) | 747/969 (77.1) |

Values are n (%). No TME grade includes TME grade missing and TME grade not assessable.

**Table S5.** Unadjusted and adjusted* Cox regression relating TME grade to local recurrence, distant recurrence, overall survival, and relative survival.

|  | Local recurrence |  | Distant recurrence | | Overall survival | | Relative survival | |
| --- | --- | --- | --- | --- | --- | --- | --- | --- |
| TME | HR (95% CI) | p | HR (95% CI) | p | HR (95% CI) | p | HR (95% CI) | p |
|  | n= 2 613 | | n= 2 613 | | n=3 915 | | | |
| Mesorectal | 1.00 |  | 1.00 |  | 1.00 |  | 1.00 |  |
| Intramesorectal | 1.90 (0.84 – 4.30) | 0.121 | 0.90 (0.62 – 1.31) | 0.571 | 0.92 (0.69 – 1.23) | 0.570 | 0.82 (0.44 – 1.53) | 0.539 |
| Muscularis propria | 3.12 (1.26 – 7.73) | 0.014 | 1.08 (0.65 – 1.77) | 0.772 | 1.10 (0.74 – 1.63) | 0.649 | 1.30 (0.63 – 2.67) | 0.482 |
| No TME grade | 1.39 (0.77 – 2.53) | 0.279 | 1.03 (0.82 – 1.28) | 0.823 | 1.05 (0.88 – 1.26) | 0.619 | 0.94 (0.65 – 1.38) | 0.783 |
|  | Adjusted* | | | | | | | |
|  | n=2 590 |  | n=2 590 |  | n= 3 881 | | | |
| Mesorectal | 1.00 |  | 1.00 |  | 1.00 |  | 1.00 |  |
| Intramesorectal | 1.94 (0.86 – 4.40) | 0.111 | 0.90 (0.61 – 1.31) | 0.565 | 0.91 (0.68 – 1.23) | 0.549 | 0.99 (0.56 – 1.74) | 0.958 |
| Muscularis propria | 3.03 (1.19 – 7.69) | 0.020 | 1.04 (0.62 – 1.72) | 0.895 | 1.02 (0.69 – 1.53) | 0.907 | 1.39 (0.70 – 2.79) | 0.346 |
| No TME grade | 1.36 (0.74 – 2.48) | 0.321 | 1.02 (0.81 – 1.28) | 0.890 | 1.02 (0.85 – 1.22) | 0.829 | 1.15 (0.81 – 1.64) | 0.438 |

*Adjusted for age, sex, BMI, clinical T stage, surgical approach (open or minimally invasive), tumour level, and preoperative treatment. No TME grade includes TME grade missing and TME grade not assessable.


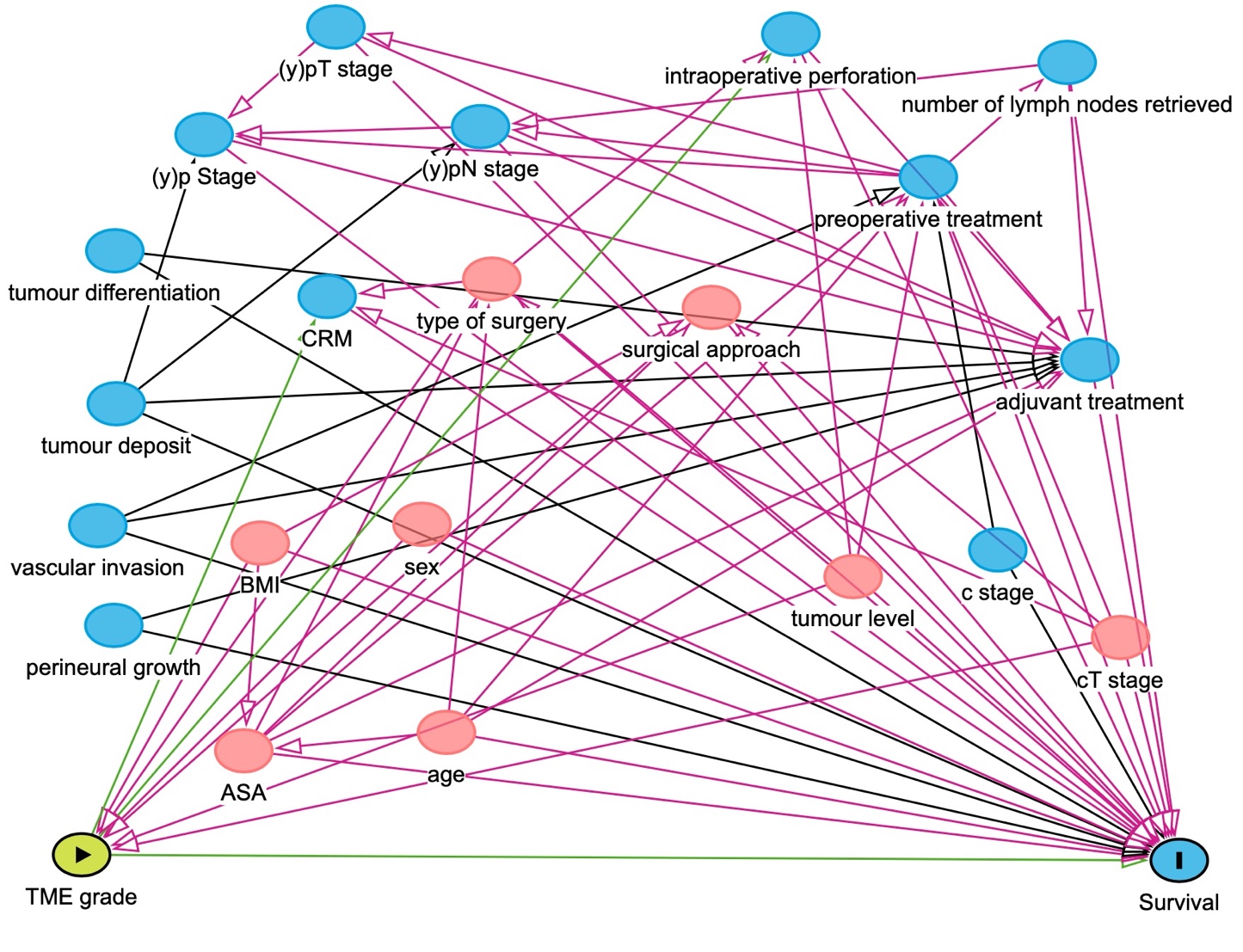


**Figure S1.** Directed acyclic graph, illustrating the assumed correlation between different variables and their potential effect on TME grade and survival. Exposure = TME grade, outcome = survival. Blue variable = ancestor of outcome, pink variable = ancestor of exposure. Green path = causal, pink path = biasing. CRM, circumferential resection margin; BMI, Body Mass Index; ASA, American Society of Anesthesiologists.


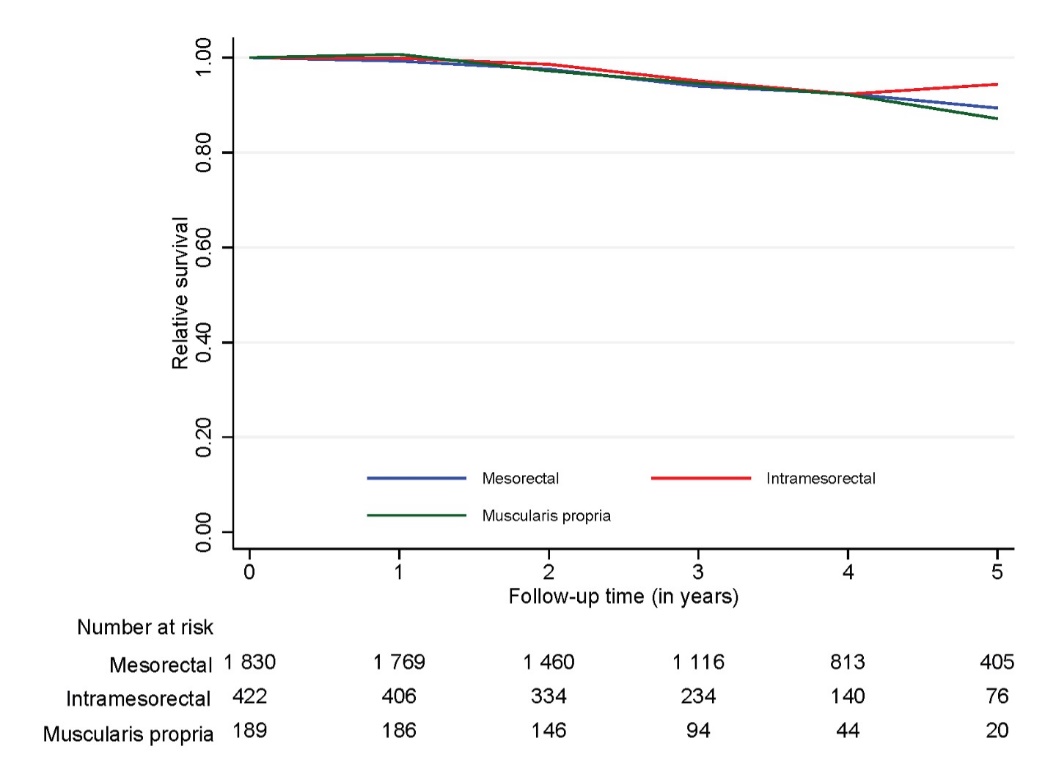


**Figure S2.** Relative survival in patients with mesorectal, intramesorectal and muscularis propria resection.
